# The association between population density and blood lipid levels in Dutch blood donors

**DOI:** 10.1186/s12942-019-0167-y

**Published:** 2019-02-04

**Authors:** Rosa de Groot, Jody C. Hoenink, Joreintje D. Mackenbach, Nicole R. den Braver, Maria G. M. Pinho, Darshan Brassinga, Femmeke J. Prinsze, Tiffany C. Timmer, Wim L. A. M. de Kort, Johannes Brug, Katja van den Hurk, Jeroen Lakerveld

**Affiliations:** 1 Department of Donor Medicine - Donor Studies, Sanquin Research, Plesmanlaan 125, 1066 CX Amsterdam, The Netherlands; 2 Department of Epidemiology and Biostatistics, Amsterdam Public Health Research Institute, Amsterdam UMC, Vrije Universiteit Amsterdam, de Boelelaan 1089A, 1081 BT Amsterdam, The Netherlands; 3 Landsteiner Laboratory, Amsterdam UMC – Location AMC, University of Amsterdam, Meibergdreef 9, 1105 AZ Amsterdam, The Netherlands; 4 Department of Public Health, Academic Medical Center Amsterdam UMC – Location AMC, University of Amsterdam, Meibergdreef 9, 1105 AZ Amsterdam, The Netherlands; 5 Amsterdam School of Communication Research (ASCoR), University of Amsterdam, Nieuwe Achtergracht 166, 1018 WV Amsterdam, The Netherlands; 6 Julius Centre for Health Sciences and Primary Care, University Medical Centre Utrecht, Universiteitsweg 100, 3584 CG Utrecht, The Netherlands; 7 Faculty of Geosciences, Utrecht University, Princetonlaan 8a, 3584 CB Utrecht, The Netherlands

**Keywords:** Population density, Blood lipid levels, Adults, Physical activity, Sedentary behavior

## Abstract

**Background:**

In low and middle-income countries (LMIC), the total and LDL cholesterol and triglyceride levels of residents of urban areas are reported to be higher than those of rural areas. This may be due to differences in lifestyle behaviors between residents of urban areas and rural areas in LMIC. In this study, our aims were to (1) examine whether or not LDL cholesterol, total/HDL ratios and triglyceride levels of individuals in densely populated areas are higher than those of individuals living in less-densely populated areas in a high-income country (HIC) and (2) investigate the potential mediating roles of physical activity and sedentary behavior.

**Methods:**

We used cross-sectional data from 2547 Dutch blood donors that participated in Donor InSight-III. Linear regression was used to analyze the association between population density and LDL cholesterol, total/HDL cholesterol ratio and HDL cholesterol. The mediating roles of moderate-to-vigorous physical activity (MVPA) and sedentary behavior were investigated in a subsample (n = 740) for which objectively measured MVPA/sedentary behavior data was available. Multiple mediation with linear regression analyses were performed and the product-of-coefficients method was used to calculate direct and indirect effects.

**Results:**

Mean LDL cholesterol and median total cholesterol/HDL cholesterol ratio and triglyceride levels were 2.89, 3.43 and 1.29 mmol/L, respectively. Population density was not associated with LDL cholesterol [β 0.00 (− 0.01; 0.01)], log transformed total/HDL cholesterol ratio [β 1.00 (1.00; 1.00)] and triglyceride levels [β 1.00 (0.99; 1.00)]. No statistically significant direct or indirect effects were found.

**Conclusion:**

Contrary to previous findings in LMIC, no evidence was found that population density is associated with blood lipid levels in blood donors in the Netherlands or that MVPA and sedentary behavior mediate this association. This may be the result of socioeconomic differences and, in part, may be due to the good health of the study population and the relatively high population density in the Netherlands. Also, compared to LMIC, differences in physical activity levels in more versus less populated areas may be less pronounced in HIC.

**Electronic supplementary material:**

The online version of this article (10.1186/s12942-019-0167-y) contains supplementary material, which is available to authorized users.

## Background

Elevated blood lipid levels are generally seen as a risk factor for cardiovascular disease and are, therefore, a key target of preventative actions [1, 2]. Despite the recommended individual-level strategies for the prevention and treatment of cardiovascular disease, including medication therapy and the promotion of healthier lifestyle behaviors, elevated blood lipid levels and the diseases associated with it are still highly prevalent and are estimated to have been responsible for 17 million deaths globally in 2013 [3, 4].

Research has shown that residential context (i.e. the place where a person lives) is an ‘upstream’ determinant of health behavior and disease outcomes [5–7]. In a recent systematic review, we identified consistent associations between living in an urban area and having higher total cholesterol (TC), low density lipoprotein (LDL) cholesterol and triglyceride levels [8]. The studies included in this review were mainly conducted in low and middle-income countries (LMIC) and the patterns observed may have been attributable to the sedentary and inactive lifestyle associated with life in urban areas in LMIC. It is well known that moderate-to-vigorous physical activity (MVPA) has a positive effect on blood lipid profiles, i.e. it increases high density lipoprotein (HDL) cholesterol levels and decreases LDL cholesterol and triglyceride levels [9–12], by means of the maturation of HDL and increased blood lipid consumption by muscle tissue during exercise [13]. There is also evidence that increased sedentary time is associated with less healthy blood lipid levels [11, 14].

Although it is likely that the physical environment influences how much we sit, evidence of a link between environmental factors and sedentary behavior has been mixed [15–17]. Also, urban areas in high-income countries (HIC) are, in general, more walkable and offer more facilities for leisure time and transport-related physical activity than urban areas in LMIC [18, 19]. Indeed, the findings of the three studies from HIC included in our review were inconclusive with regard to the association between urbanization and blood lipid levels [20–22]. As such, further investigation of whether or not urban versus rural differences in blood lipid levels can be explained by levels of physical activity and sedentary behavior in HIC is warranted [9, 23].

It may be the case that adults in LMIC who live in rural areas have less access to cars, have more physically demanding occupations and/or are exposed less often to foods that are high in energy, salt, sugar and fat, making a more active lifestyle and healthier diet more likely in these individuals as compared to adults from rural areas in HIC. In HIC, physical activity levels are often higher in urban areas as compared to rural areas [24, 25]. There is, thus, reason to question whether or not blood lipid levels are also less healthy, i.e. with higher LDL cholesterol, TC/HDL ratio and triglycerides (TG) levels, in individuals residing in areas with higher population density in HIC. Differences in health outcomes, such as greater incidence and/or risk of type 2 diabetes and cancer in urban residents and lower incidence of obesity in rural residents, have been reported in literature [26–29]. Gaining insight into the health consequences of urbanization is essential, as much as 70% of the global population is projected to reside in urban areas by 2050 [30, 31]. Most studies conducted to date have not included data from the more or most affluent countries and regions, and there is reason to believe that an association between urbanization and blood lipid levels may differ according to level of affluence. In light of this, our study provides valuable information from a high-income country context.

To contribute to the evidence-base on rural–urban differences in HIC, this study aims to investigate the association between population density and blood lipid levels in blood donors across the Netherlands. We hypothesize that LDL cholesterol, TC/HDL ratios, and TG levels are higher in individuals who reside in more densely populated areas. In addition, the potential mediating role of physical activity and sedentary behavior is investigated.

## Methods

### Study design and population

DIS is a Dutch cohort study of blood and plasma donors carried out by Sanquin—the only organization in the Netherlands authorized to collect blood from donors [32]. The wide geographical distribution of Sanquin’s blood collection centers across the Netherlands and the large number of blood donors involved in the study yield data that allows us to explore the association of population density on lifestyle behaviors and blood lipid levels.

**Fig. 1:**
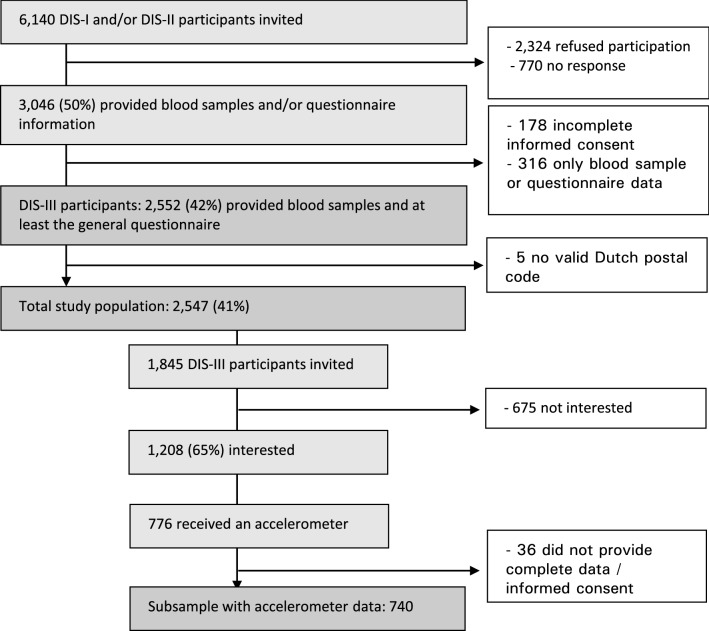
Flow chart of total study population and subsample with accelerometer data

This study is a cross-sectional analysis of data from the third data collection wave of the Donor InSight study (DIS-III; April 2015 and December 2016). Eligibility criteria for DIS-III were 1) participation in one or both of the first two data collection waves of DIS and 2) a registered hemoglobin measurement from the donor’s first donation at Sanquin. A total of 6140 donors were invited to participate in DIS-III (see Fig. 1 for a complete breakdown of numbers). Of those, 3046 participants (50%) provided questionnaire information and/or a blood sample. For the main analyses, we excluded those donors who (1) did not provide complete informed consent (n = 178); (2) did not complete both the general questionnaire and provide a blood sample (n = 316) or (3) for which we did not have valid geographical information (i.e. a Dutch postal code) (n = 5). The total study population consisted of 2547 participants. Of those 2547 DIS-III participants, 1845 (Fig. 1) were invited via an extra question in the general questionnaire to wear an accelerometer for seven consecutive days during waking hours in order to objectively measure physical activity and sedentary behavior. As the accelerometer study was set up after the main DIS-III study, not all participants received an invitation. Two thirds (66%) of the DIS-III participants who received the invitation were interested in participating (n = 1208) and 776 participants were sent an accelerometer by mail. Of these, 36 did not provide valid data, mainly due to lack of interest, time constraints and technical issues. This resulted in a subsample with objectively measured physical activity and sedentary behavior for 740 participants.

The Medical Ethical Committee of the Academic Medical Center Amsterdam, the Netherlands, approved DIS-III and all participants gave their written, informed consent.

## Measurements

### Blood lipid levels

Primary outcome measures for this study were LDL cholesterol, total cholesterol/high density lipoprotein cholesterol ratio (TC/HDL ratio) and triglyceride levels. Although there is some conflicting evidence in the literature as to what constitutes ‘favorable’ blood lipid levels [33, 34], throughout this article we consistently refer to low LDL cholesterol, low TC/HDL ratio and triglyceride levels as ‘favorable blood lipid levels’. Non-fasting, whole blood samples were collected in 3 mL lithium heparin tubes from the diversion pouch or through venipuncture if a donor was not able or willing to make a full donation. The first 20–30 mL of a donation is collected in the diversion pouch and is routinely used for screening and blood-typing purposes [35]. TC, HDL cholesterol and triglycerides were determined using enzymatic colorimetric methods (Roche/Hitache Cobas C, Basel, Switzerland). To calculate LDL cholesterol levels, the Friedewald formula was used: TC − HDL cholesterol − (TG/2.2) [36].

### Population density

Participants’ 6 digit postal codes were linked to neighborhood population density (number of inhabitants per km^2^), as registered by Statistics Netherlands (CBS) in 2014, as a proxy for the degree of urbanization. Population density is expressed in 1000 inhabitants per km^2^. While exposure to urban or rural environments is not fully encompassed by considering residential area alone, individuals are influenced by the place in which they live [37]. Diez Roux et al. [37] state that ‘macro-level’ factors such as the dependence on automobiles for transport, perceived safety, marketing of (un)healthy foods and (lack of) regulation of unhealthy products exist at the neighborhood level and thereby have a direct influence on individuals. In addition, a focus on administrative neighborhoods allows for the possible future implementation of policy.

### Physical activity and sedentary behavior

MVPA and sedentary behavior were studied as potential mediating variables that could account for the association between population density and blood lipid levels. Sedentary behavior was defined as any waking behavior with an energy expenditure ≤ 1.5 metabolic equivalents (METs), while in a sitting, reclining or lying posture [38]. Accelerometers (wGT3X-BT and GT3X Actigraph, Pensacola, USA) were used to objectively measure both MVPA and sedentary behavior. MVPA and sedentary behavior were classified according to Troiano Adult (2008) cut-off points [39] > 3 MET and ≤ 1.5 MET, respectively, and are reported as mean minutes per day. To calculate mean minutes per day, the total number of minutes per category (MVPA and sedentary behavior) were divided by the number of valid days. A minimum of four valid days were required for the analysis and a day was considered valid if the wear time was at least ten hours. These measurements took place as close as possible, in time, to the DIS-III blood sampling.

MVPA and sedentary behavior were also assessed by self-reporting for sensitivity analyses in the entire study population using the short version of the International Physical Activity Questionnaire (IPAQ) [40]. The IPAQ scoring protocol was used to clean the data and calculate MVPA and sedentary behavior.

### Co-variates

Information on socio-demographic factors, lifestyle behaviors and medication use was obtained on the basis of self-administrated questionnaires. Socio-demographic variables included age, sex and level of education as a proxy for socio-economic status. Higher education was defined as having completed tertiary education, i.e. college or university, and lower education was defined as having only completed education below tertiary education. Smoking was categorized as current or previous/never. Consumption of alcoholic beverages consisted of ‘yes’ and ‘no’ as answer possibilities. Medication use was classified as the use of lipid-modifying medication according to the WHO Anatomical Therapeutic Classification (ATC, code *C10 Lipid*-*modifying agents*) into ‘yes’ or ‘no’ [41].

### Statistical analyses

Descriptive statistics are presented as mean ± standard deviation or, in the event of a skewed distribution, as median and interquartile range (IQR). The underlying assumptions of linear regression analysis were met. The data was checked prior to statistical analysis and right-skewed data were log-transformed. Missing data per variable ranged from 0.2% (HDL cholesterol and TC) to 14.3% (self-reported MVPA) with a total of 72% complete cases and 3% missing values. Missing data were assumed to be missing at random and multiple imputation using predictive mean matching on item score level was performed. A total of 30 imputed data sets were constructed as recommended by White et al. [42]. All variables used in the analyses were imputed.

Population density and blood lipid levels

We assessed the association between population density and blood lipid levels using multiple linear regression analysis (n = 2547). We investigated effect modification by age and sex by adding interaction terms between population density and age and sex to the regression models. We present the results of unadjusted models; models adjusted for age and sex; those additionally adjusted for alcohol consumption and smoking; those additionally adjusted for educational level; and those additionally adjusted for the use of lipid-modifying medication.

Mediation by MVPA and sedentary behavior

**Fig. 2:**
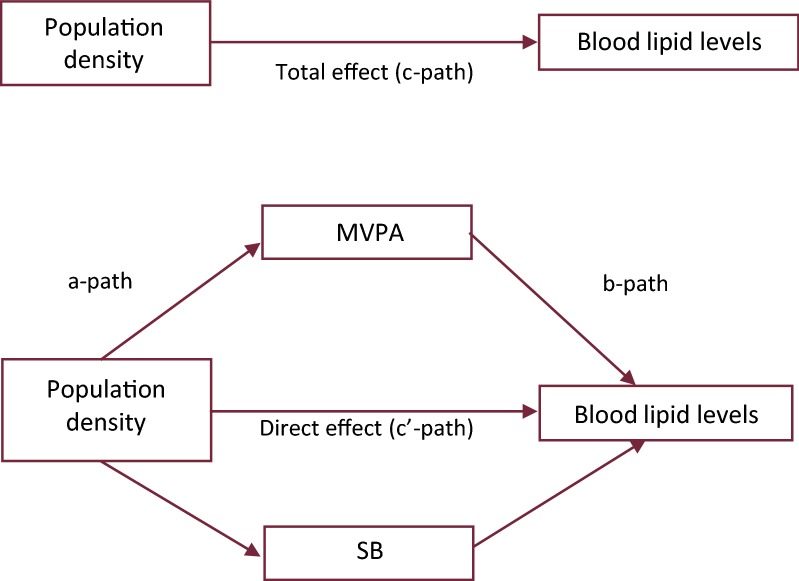
Mediation analysis framework. *c path* total effect of population density on blood lipid levels, *c′ path* direct effect of population density on blood lipids adjusted for mediating variable, *a*-*path* effect of population density on mediator, *b*-*path* effect of mediator on blood lipids, *a*-*path *×* b*-*path* indirect effect of population density on blood lipid levels through mediating variables

To examine mediation by time engaged in MVPA and sedentary behavior of the association between population density and blood lipid levels, we conducted formal multiple mediation analyses in the subsample (n = 740) with objectively measured data on MVPA and sedentary behavior. Multiple linear regression analysis using the macro PROCESS 3.0 for SPSS was used to conduct the mediation analysis [43]. The PROCESS output is comprised of three linear regression models. The first model estimates the total effect (c-path) of population density on blood lipid levels. The second model estimates the association between population density and MVPA/sedentary behavior (a-path) and the third and final model estimates both the direct effect (c′-path) of population density on blood lipid levels and the association between MVPA/sedentary behavior on blood lipid levels (b-path). The indirect effect was calculated as the product of the a and b path with a 95% bootstrapped confidence interval, based on 5000 bootstrap resamples, see Fig. 2. Rubin’s rules were used to pool all coefficients and 95% confidence intervals (95% CI) of the a, b and c-paths and indirect effect. The proportion mediated was calculated using Eq. 1, only if (1) significant mediation was found; (2) the total (c path) and indirect effect had the same direction and (3) the indirect effect was smaller than the total effect [43].

1 $$ Proportion\,mediated = \frac{a*b}{{a*b + c^{{\prime }} }} $$

where in Eq. 1 $a$ represents the effect of the exposure variable $X$ on the mediator variable *M*, $b$ represents the effect of mediator variable $M$ on the outcome variable $Y$ and $c^{{\prime }}$ represents the direct effect of the exposure variable $X$ on the outcome variable $Y$.

As a sensitivity analysis, we repeated (1) the analyses with non-imputed data and (2) the mediation analyses with self-reported MVPA and sedentary behavior data in the total study population.

## Results

Table 1 describes donor characteristics for the total study population (n = 2547), as well as for participants for whom accelerometer data was available (i.e. the subsample used for the mediation analyses, n = 740). Both groups were similar with regard to socio-demographic variables: about 45% were male and self-reported smoking was around 8.5% for the both groups. Blood lipid levels were similar in the total study population as compared to the subsample: 2.89 ± 0.84 mmol/L versus 2.92 ± 0.84 mmol/L total cholesterol; 1.29 (0.93–1.81) mmol/L versus 1.27 (0.93–1.74) mmol/L. Median (IQR) population density of the total study population was 4043 inhabitants (2055–6202) per km^2^.

**Table 1:** Characteristics of total Donor InSight (DIS)-III study population and subsample with accelerometer data

	Total DIS-III population	Subsample of DIS-III with accelerometer data
	n = 2547	n = 740
Sex (n (%) males)	1143 (45)	338 (46)
Age (years)	48 ± 13	50 ± 13
Education		
Low (n (%))	1636 (64)	478 (65)
High (n (%))	899 (35)	259 (35)
IPAQ		
Sedentary behavior (minutes per day)	480 (300–660)	480 (300–600)
MVPA (minutes per day)	51 (21–113)	51 (21–119)
150 min or more per week MVPA (n (%))	1631 (64)	492 (66)
Accelerometer		
Sedentary behavior (minutes per day)	–	549 ± 87
MVPA (minutes per day)	–	29 (19–45)
150 min or more per week MVPA (n (%))	–	492 (66)
Current smoker (n (%))	201 (9)	62 (8)
Alcohol consumption (yes) (n (%))	2067 (81)	609 (82)
Lipid modifying medication (yes) (n (%))	142 (6)	43 (6)
Blood lipids		
Total cholesterol (mmol/L)	5.05 ± 0.98	5.11 ± 0.97
HDL cholesterol (mmol/L)	1.49 ± 0.40	1.53 ± 0.42
LDL cholesterol (mmol/L)	2.89 ± 0.84	2.92 ± 0.84
Triglycerides (mmol/L)	1.29 (0.93–1.81)	1.27 (0.93–1.74)
TC/HDL ratio (mmol/L)	3.43 (2.80–4.18)	3.37 (2.78–4.14)

Binary data are shown as numbers (%) and continuous data are shown as mean ± standard deviation or as median (interquartile range) in case of skewed data

*MVPA* moderate to vigorous physical activity, *HDL cholesterol* high density lipoprotein cholesterol, *LDL cholesterol* low density lipoprotein cholesterol, *TC/HDL cholesterol* total cholesterol/high density lipoprotein cholesterol ratio, *IPAQ* International Physical Activity Questionnaire

Population density and blood lipid levels

Table 2 shows the regression coefficients of the analyses of population density and LDL cholesterol, TC/HDL ratio and TG levels in the total study population. As no evidence of effect modification was found, all analyses were adjusted for age, sex, smoking, alcohol consumption and the use of lipid-modifying medication. Population density was not associated with any of the blood lipid level levels LDL cholesterol [β 0.00 (− 0.01; 0.01)], log transformed TC/HDL ratio [β 1.00 (1.00; 1.00)] and log transformed triglyceride levels [β 1.00 (0.99; 1.00)].

**Table 2:** Association between population density and blood lipid levels based on linear regression analyses

Dependent variable	Model 1	Model 2	Model 3	Model 4	Model 5
	β or RR 95% CI	β or RR 95% CI	β or RR 95% CI	β or RR 95% CI	β or RR 95% CI
LDL cholesterol	− 0.01 (− 0.02, 0.00)	0.00 (− 0.01, 0.01)	0.00 (− 0.01, 0.01)	0.00 (− 0.01, 0.01)	0.00 (− 0.01, 0.01)
TC/HDL cholesterol ratio^a^	0.99 (0.99, 1.00)	1.00 (1.00, 1.00)	1.00 (0.99, 1.00)	1.00 (0.99, 1.00)	1.00 (1.00, 1.00)
Triglycerides^a^	0.99 (0.99, 1.00)	1.00 (0.99, 1.00)	1.00 (0.99, 1.00)	1.00 (0.99, 1.00)	1.00 (0.99, 1.00)

Model 1: population density and blood lipid levels. Model 2: model 1 + sex and age. Model 3: model 2 + education. Model 4: model 3 + smoking and alcohol consumption. Model 5: model 4 + lipid modifying medication

*β* unstandardized regression coefficient, *95%CI* 95% confidence interval, population density is expressed per 1000 s of inhabitants per km^2^, *LDL* low density lipoprotein cholesterol, *TC/HDL* total cholesterol/high density lipoprotein cholesterol ratio, *TG* triglycerides

^a^Residuals of TC/HDL and TG were not normally distributed and were therefore log transformed, this table presents log transformed data

Mediation models

Table 3 shows the adjusted mediation models of population density and the three blood lipid levels for objectively measured MVPA and sedentary behavior. The associations between population density and MVPA or sedentary behavior were not significant (a-path). Higher levels of MVPA were significantly associated with lower TC/HDL ratio and triglyceride levels (b-path). The coefficient of − 0.43 indicates that an increase of 10 min MVPA is associated with a relative decrease in triglyceride level of 0.43 mmol/L.

**Table 3:** Association between population density and blood lipids and mediation by objectively measured MVPA and SB adjusted for confounders

IV	DV	MV	Effect of population density on lifestyle behaviors	Effect of lifestyle behaviors on blood lipids	Total effect	Direct effect	Indirect effect
			(a-path)	(b-path)	(c-path)	(c′-path)	(a-path × b-path)
			β 95% CI	β 95% CI	β 95% CI	β 95% CI	β 95% BCI
Population density per 1000 inhabitants	LDL cholesterol	MVPA	0.00 (− 0.00, 0.00)	− 0.16 (− 0.91, 0.59)	0.00 (− 0.02, 0.01)	0.00 (− 0.02, 0.01)	0.00 (0.00, 0.00)
		SB	0.00 (− 0.17, 0.16)	0.00 (0.00, 0.01)			
	TC/HDL cholesterol ratio	MVPA	0.00 (− 0.00, 0.00)	− *0.43 (*− *0.71,* − *0.16)*	0.00 (− 0.01, 0.00)	0.00 (− 0.01, 0.00)	0.00 (0.00, 0.00)
		SB	0.00 (− 0.17, 0.16)	0.00 (0.00, 0.00)			
	Triglycerides	MVPA	0.00 (− 0.00, 0.00)	− *0.55 (*− *1.02,* − *0.08)*	0.00 (− 0.01, 0.01)	0.00 (− 0.01, 0.01)	0.00 (0.00, 0.00)
		SB	0.00 (− 0.17, 0.16)	0.00 (− 0.01, 0.00)			

Residuals of MVPA, TC/HDL and TG were not normally distributed and were, therefore, log transformed, this table presents log transformed data. italics regression coefficients and 95% confidence intervals are statistically significant p < 0.05

*IV* Independent variable, *DV* dependent variable, *MV* mediating variable, *a*-*path* association between population density and mediating variable, *b*-*path* association between mediating variable and blood lipid outcome, *c*-*path* association between population density and blood lipid outcome, *c′*-*path* association between population density and blood lipid outcome adjusted for mediating variables, *indirect effect* indirect effect of population density on blood lipid outcome through mediating variables, *β* unstandardized regression coefficient, *95% CI* 95% confidence interval, *BCI* bootstrapped confidence interval, *SB* sedentary behavior expressed per 10 min, *MVPA* moderate to vigorous physical activity expressed per 10 min, *TC/HDL cholesterol ratio* total cholesterol/high density lipoprotein cholesterol ratio, *LDL* low density lipoprotein cholesterol, *TG* triglycerides

For all models, the direct paths (c′-path) of population density and blood lipids levels through MVPA and sedentary behavior were not significantly associated, neither were the indirect paths (a-path × b-path). No evidence was found for a mediating role of MVPA and sedentary behavior.

Overall, sensitivity analyses with the non-imputed data generated similar results (Additional file 1: Tables S1 and S2). Mediation analyses with self-reported MVPA and sedentary behavior in the study population (n = 2547) yielded similar results (Additional file 1: Table S3).

## Discussion

In this study of blood donors in the Netherlands, we examined whether or not blood lipid levels of residents of urban areas had a healthier profile than those of rural residents and investigated the potential mediating role of physical activity and sedentary behavior in this association. No significant or otherwise meaningful associations between population density and blood lipid levels were found. No evidence was found for the existence of a mediating role of MVPA or sedentary behavior in the association between population density and blood lipid levels. We did, however, find an association between objectively measured MVPA and more favorable blood lipid levels.

Our findings regarding the association between population density and blood lipid levels differ from the findings of previous studies, largely conducted in LMIC [8]. The absence of an association between population density and blood lipid levels may be explained by several factors, including differences between HIC and LMIC, the absence of information on either the food environment or food intake, the operationalization of urbanization used and the population under study. We also did not find any association between population density and MVPA, in spite of the fact that more compact and densely populated urban areas are often hypothesized to be living spaces that are facilitate physical activity due to the relative closeness of amenities, workplaces, etc. [44, 45]. However, our finding that higher MVPA levels were associated with more favorable blood lipid levels is consistent with the results of other studies [9, 10, 46].

Several relevant differences between LMIC and HIC have been identified in the literature. First, while residing in an urban area is associated with lower levels of physical activity in LMIC, it is associated with higher physical activity levels in HIC [24, 25]. Secondly, the differences in terms of food environment in more and less densely populated areas in HIC is likely to be less than it is in LMIC, especially in LMIC that are undergoing a food transition [47]. While in rural areas in LMIC the food environment remains relatively stable, changes in the food environment in densely populated areas in LMIC take place at a rapid pace, a pace that is unlikely to be achieved in the Netherlands [47, 48]. Thirdly, urban–rural differences in occupational physical activity are likely to be smaller in HIC as compared to LMIC, as a significant share of labor in HIC, for both urban and rural residents, involves desk-based, seated work [49, 50]. More physically demanding labor may be expected in rural areas in LMIC, especially in very remote areas. Nonetheless, the majority (66%, based on the accelerometer subsample) of our study population adhered to the physical activity guidelines of 150 min of more per week, which may have resulted in limited variation in physical activity in the study population [51].

Another factor that could explain the absence of a relevant association could be that urbanization is operationalized differently across various studies and this heterogeneity is likely to influence results and make replication, interpretation and extrapolation of findings challenging [6, 52]. Population density does not directly capture other environmental characteristics associated with lifestyle behaviors such as infrastructure, availability and density of (fast-) food restaurants, access to healthcare and safety. Neighborhoods with low population density levels by Dutch standards may be regarded as high-density or urban areas in other countries. One might speculate that an area with low population density in the Netherlands provides more opportunities to be physically active than a comparable area in a LMIC. It could be that in the Netherlands, even an area with relatively low population density may have already reached a certain threshold after which there are no measureable differences in terms of effect. Despite the wide geographical distribution of our study population throughout the Netherlands, the median population density of place of residence of the study population was high [4043 inhabitants (2055–6202) per km^2^] as compared to the 2016 mean population density of 504 inhabitants per km^2^. This fairly ‘urban’ study population may have made it more difficult to identify differences.

There are, however, other possible explanations as to why no association was found between population density and blood lipid levels. Our study population was relatively healthy, with respect to both blood lipid levels and physical activity, which is probably the result of donor selection based on eligibility criteria and self-selection [53–56]. Beneficial effects of repeated blood donation on blood lipid profiles have been suggested in the literature [57, 58]. However, in these studies, active donors were compared with new and former donors [57, 58]. As such, self-selection of donors may have caused considerable confounding. If they exist, the beneficial effects of blood donation would be similar across the study population regardless of the area of residence and would only have yielded less variation. The selection of relatively healthy donors may have led to a weakening of associations, as greater variation in blood lipid levels may have resulted in greater contrasts between donors. However, it may be argued that since the effect sizes are all close to zero in this study, effect sizes in the general population—if any—are likely to be quite small.

Strengths and limitations

This study is among the first to investigate the association between population density and blood lipid levels in a HIC. The strengths of this study include the our use of objectively measured data on physical activity and sedentary behavior, which allowed us to study the hypothesized mechanism—via MVPA and sedentary behavior—through which population density was hypothesized to be associated with blood lipid levels. Furthermore, most previous studies dichotomize the urbanization variable as urban versus rural, which limits our understanding of whether it is relative urbanization level, population density in a country, or a certain absolute population density that is associated with blood lipid levels. The results of our mediation analyses must be interpreted with caution, as these were conducted using cross-sectional data. Furthermore, while food intake is an important determinant of blood lipid levels, we had no information on actual intake of food high in fat and/or sugar, while both are related to blood lipid levels and cardiovascular disease [59, 60]. Although it was an aim of this study to link population density of residential neighborhoods to blood lipid levels, this inherently omits individuals’ exposure to other potentially relevant areas such as the workplace, the area covered during commuting and the leisure environment.

Future studies could seek to replicate these findings in other HIC in a general population, preferably in countries or geographical areas (e.g. provinces, regions) with more variation in terms of population density. We also recommended assessing other aspects of exposure associated with population density, including the food environment.

In summary, we found no evidence that population density and blood lipid levels in blood donors in the Netherlands were associated in any significant or meaningful way, nor did we find any indication that MVPA or sedentary behavior mediated this association. This study suggests that the association between population density and blood lipid levels might be different in HIC than it is in LMIC.

## Additional file

**Additional file 1: Table S1.** Associations between population density and blood lipid levels based on linear regression analyses in non-imputed data. **Table S2**. Associations between population density and blood lipids and mediation by objectively measured MVPA and SB in non-imputed data. **Table S3.** Associations between population density and blood lipids and mediation by self-reported MVPA and SB.
