# Supplementary material for: The association between population density and blood lipid levels in Dutch blood donors
Source: Int J Health Geogr. 2019 Feb 4;18:3. doi: 10.1186/s12942-019-0167-y (PMC6360723; doi:10.1186/s12942-019-0167-y)
Supplement: Supplementary file 1 — Additional file 1: Table S1. Associations between population density and blood lipid levels based on linear regression analyses in non-imputed data. Table S2. Associations between population density and blood lipids and mediation by objectively measured MVPA and SB in non-imputed data. Table S3. Associations between population density and blood lipids and mediation by self-reported MVPA and SB. [file 12942_2019_167_MOESM1_ESM.docx]

# Additional files

|  | Model 1 | Model 2 | Model 3 | Model 4 | Model 5 |
| --- | --- | --- | --- | --- | --- |
| Dependent variable | β or RR 95%CI | β or RR 95%CI | β or RR 95%CI | β or RR 95%CI | β or RR 95%CI |
| LDL cholesterol | -0.01 (-0.02 , 0.00) | 0.00 (-0.01 , 0.01) | 0.00 (-0.01 , 0.01) | 0.00 (-0.01 , 0.01) | 0.00 (-0.01 , 0.01) |
| TC/HDL cholesterol ratio | 1.00 (0.99 , 1.00) | 1.00 (0.99 , 1.00) | 1.00 (1.00, 1.00) | 1.00 (1.00 , 1.00) | 1.00 (1.00 , 1.00) |
| Triglycerides | 0.99 (0.99 , 1.00) | 1.00 (0.99 , 1.00) | 1.00 (0.99 , 1.00) | 1.00 (0.99 , 1.00) | 1.00 (0.99 , 1.00) |

| Model 1: population density and blood lipid levels. Model 2: model 1 + sex and age. Model 3: model 2 + education. Model 4: model 3 + smoking and alcohol consumption. Model 5: model 4 + lipid modifying medication. β: unstandardized regression coefficient, 95%CI: 95% confidence interval, population density is expressed per 1000s of inhabitants per km^2^. LDL: low density lipoprotein cholesterol, TC/HDL: total cholesterol/high density lipoprotein cholesterol ratio, TG: triglycerides. ^a^Residuals of TC/HDL and TG were not normally distributed and were therefore log transformed, this table presents log transformed data. |
| --- |

**Additional file Table S1 Associations between population density and blood lipid levels based on linear regression analyses in non-imputed data**

**Additional file Table S2 Associations between population density and blood lipids and mediation by objectively measured MVPA and SB adjusted for confounders in non-imputed data**

| IV | DV | MV | Effect of population density on lifestyle behaviors  (a-path)  β 95%CI | Effect of lifestyle  behaviors on blood lipids  (b-path)  β 95%CI | Total effect  (c-path)  β 95%CI | Direct effect  (c’-path)  β 95% CI | Indirect effect  (a-path x b-path)  β 95%BCI |
| --- | --- | --- | --- | --- | --- | --- | --- |
| Population density  per 1000 inhabitants | LDL cholesterol | MVPA SB | 0.00 (0.00 , 0.00) 0.01 (-0.16 , 0.19) | -0.15 (-0.96 , 0.67) 0.00 (0.00 , 0.01) | 0.00 (-0.02 , 0.01) | 0.00 (-0.02 , 0.01) | 0.00 (0.00 , 0.00) |
|  | TC/HDL cholesterol ratio | MVPA SB | 0.00 (0.00 , 0.00) 0.01 -0.16 , 0.19) | **-0.40 (-0.69 , -0.10)** 0.00 (0.00 , 0.00) | 0.00 (-0.01 , 0.01) | 0.00 (-0.01 , 0.01) | 0.00 (0.00 , 0.00) |
|  | Triglycerides | MVPA SB | 0.00 (0.00 , 0.00) 0.01 (-0.16 , 0.19) | **-0.52 (-1.03 , -0.01)** 0.00 (-0.01 , 0.00) | 0.00 (-0.01 , 0.01) | 0.00 (-0.01 , 0.01) | 0.00 (0.00 , 0.00) |

IV: Independent variable, MV: mediating variable, DV: dependent variable, c*-*path: association between population density and blood lipid outcome, c’-path: association between population density and blood lipid outcome adjusted for mediating variable, a-path: association between population density and mediating variable, b-path: association between mediating variable and blood lipid outcome, indirect effect: indirect effect of population density on blood lipid outcome through mediating variable. β: unstandardized regression coefficient, BCI: bootstrapped confidence interval, SB: sedentary behavior expressed per 10 minutes, MVPA: moderate to vigorous physical activity expressed per 10 minutes, TC/HDL: total cholesterol/high density lipoprotein cholesterol ratio, LDL: low density lipoprotein cholesterol, TG: triglycerides. Residuals of MVPA, TC/HDL and TG were not normally distributed and were therefore log transformed, data presented in this table shows log transformed data. **Bold** regression coefficients and 95% confidence intervals are statistically significant p<0.05.

**Additional file Table S3 Associations between population density and blood lipids and mediation by self-reported MVPA and SB adjusted for confounders**

| IV | DV | MV | Effect of population density on lifestyle behaviors  (a-path)  β 95%CI | Effect of lifestyle  behaviors on blood lipids  (b-path)  β 95%CI | Total effect  (c-path)  β 95%CI | Direct effect  (c’-path)  β 95% CI | Indirect effect  (a-path x b-path)  β 95%BCI |
| --- | --- | --- | --- | --- | --- | --- | --- |
| Population density  per 1000 inhabitants | LDL cholesterol | MVPA SB | 0.00 (0.00 , 0.00) 0.27 (-0.82 , 1.36) | **-0.27 (-0.45 , -0.08)** 0.00 (0.00 , 0.00) | 0.00 (-0.01 , 0.01) | 0.00 (-0.01 , 0.01) | 0.00 (0.00 , 0.00) |
|  | TC/HDL cholesterol ratio | MVPA SB | 0.00 (0.00 , 0.00) 0.27 (-0.82 , 1.36) | **-0.17 (-0.24 , -0.11)** 0.00 (0.00 , 0.00) | 0.00 (0.00 , 0.00) | 0.00 (0.00 , 0.00) | 0.00 (0.00 , 0.00) |
|  | Triglycerides | MVPA SB | 0.00 (0.00 , 0.00) 0.27 (-0.82 , 1.36) | **-0.23 (-0.34 , -0.12)** 0.00 (0.00 , 0.00) | 0.00 (-0.01 , 0.00) | 0.00 (-0.01 , 0.00) | 0.00 (0.00 , 0.00) |

IV: Independent variable, DV: dependent variable, MV: mediating variable, a-path: association between population density and mediating variable, b-path: association between mediating variable and blood lipid outcome, c-path: association between population density and blood lipid outcome, c’-path: association between population density and blood lipid outcome adjusted for mediating variable, indirect effect: indirect effect of population density on blood lipid outcome through mediating variable. β: unstandardized regression coefficient, BCI: bootstrapped confidence interval, SB: sedentary behavior expressed per 10 minutes, MVPA: moderate to vigorous physical activity expressed per 10 minutes, TC/HDL: total cholesterol/high density lipoprotein cholesterol ratio, LDL: low density lipoprotein cholesterol, TG: triglycerides. Residuals of MVPA, TC/HDL and TG were not normally distributed and were therefore log transformed, data presented in this table shows log transformed data. **Bold** regression coefficients and 95% confidence intervals are statistically significant p<0.05.
